# Draft Genome Sequence of *Exiguobacterium pavilionensis* Strain RW-2, with Wide Thermal, Salinity, and pH Tolerance, Isolated from Modern Freshwater Microbialites

**DOI:** 10.1128/genomeA.00597-13

**Published:** 2013-08-08

**Authors:** Richard Allen White, Christopher J. Grassa, Curtis A. Suttle

**Affiliations:** Department of Microbiology & Immunology, University of British Columbia, Vancouver, BC, Canadaa; Department of Botany, University of British Columbia, Vancouver, BC, Canadab; Department of Earth, Ocean & Atmospheric Sciences, University of British Columbia, Vancouver, BC, Canadac; Canadian Institute for Advanced Researchd ‡

## Abstract

We report the draft genome sequence of *Exiguobacterium pavilionensis* strain RW-2, isolated from a cold thrombolytic microbialite. The isolate grows at temperatures from 4 to 50°C, at pH levels from 5 to 11, and in media without added NaCl or KCl or with 7% added NaCl.

## GENOME ANNOUNCEMENT

Collins et al. first described the genus *Exiguobacterium* with the characterization of *E. aurantiacum* strain DSM6208^T^ from an alkaline potato processing plant (1). Subsequently, other members of the genus have been isolated from diverse locations, including hot spring (2, 3), hydrothermal vent (4), permafrost (5–8), ice (9), processing plant (1, 10), moraine (11), soil (12), marine (13), freshwater (14, 15), and biofilm (14, 16) environments, brine shrimp (17), and a stromatolite from an Andean lake (18, 19).

*Exiguobacterium pavilionensis* strain RW-2 grows at temperatures between 4 and 50°C and at pH levels from 5 to 11 and is halotolerant, growing in media without added NaCl or KCl or with 7% added NaCl. The source of *E. pavilionensis* strain RW-2 was a nonlayered, clotted thrombolytic microbialite from the hypolimnion of the oligotrophic (3.3 µg liter^–1^ total phosphorus) Pavilion Lake (50.86677°N 121.74191°W) near Lillooet, BC, Canada. The microbialite was also the source of *Agrococcus pavilionensis* strain RW-1 (20). The lake is slightly alkaline (pH ~8 to 8.5 ), with a high carbonate content (~140 to 193 mg CaCO_3_ liter^–1^), and is permanently cold (4°C to 8°C) in the hypolimnion (21, 22).

DNA was extracted using Qiagen QiaAMP followed by MinElute cleanup columns. The Illumina library was constructed using Lucigen’s NxSeq library prep kit without final PCR enrichment. Quality control of the resulting library was completed using Agilent high-sensitivity DNA chips and digital droplet PCR (23).

Whole-genome shotgun sequencing was completed using Illumina MiSeq in the 250-bp paired-read format. A partial flow cell obtained 7.98 million raw reads, with 1,969,307,126 bp of raw sequence. Paired reads were error corrected, extended, and gap filled using AllPaths-LG (version 44837) (24). The error-corrected reads were then screened for phiX contamination, which was determined to be 0.98% by use of Bowtie2 (version 2.1.0) (25). The phiX reads were removed using Bowtie2 (25) and Picard tools (version 1.90) (http://picard.sourceforge.net). The error-corrected and phiX-removed reads were assembled using Ray assembler (version 2.2.0), yielding 23 contigs summing to 3,019,504 bp, with an average contig length of 131,282 bp and a largest contig of 947,149 bp (*N*_50_ length, 705,844; *N*_90_ length, 98,909 bp; G+C content, 52.05%) (26, 27).

Annotation was conducted on the RAST annotation server using the Glimmer-3 option FIGfam database release-59 (28). The genome annotation contains 3,079 predicted protein-coding genes, including 50 noncoding RNAs and 371 predicted SEED subsystem features. Genes for survival within the cold oligotrophic environment of Pavilion Lake are predicted within the genome of *E. pavilionensis*. Genes putatively encoding a Pho regulon for high-affinity phosphate uptake, carbon starvation, oxidative stress, and cold-shock proteins are consistent with growth in cold oligotrophic waters.

This is the sixth genome to be sequenced from the genus *Exiguobacterium* (2, 8, 16), but the first from a thrombolytic microbialite. Although isolated from a permanently cold freshwater environment, this organism can grow at 50°C and is able to tolerate high salt concentrations and acidic and alkaline conditions. This genome will allow for comparison with other members of this genus and provide insights into the mechanisms that allow these bacteria to thrive under such wide-ranging conditions.

### Nucleotide sequence accession numbers.

This whole-genome shotgun project has been deposited at DDBJ/EMBL/GenBank under the accession number ATCL00000000. The version described in this paper is version ATCL01000000.
